# Pneumonia incidence trends in UK primary care from 2002 to 2017: population-based cohort study

**DOI:** 10.1017/S0950268819001559

**Published:** 2019-09-09

**Authors:** Xiaohui Sun, Abdel Douiri, Martin Gulliford

**Affiliations:** 1 King's College London, School of Population Health and Environmental Sciences, London, UK; 2 National Institute for Health Research Biomedical Research Centre at Guy's and St Thomas’ National Health Service Foundation Trust, London, UK

**Keywords:** Antibiotics, epidemiology, pneumonia, primary care, respiratory tract infection

## Abstract

Increasing hospital admissions for pneumonia have been reported recently but it is not known whether pneumonia incidence rates have increased in the community. To determine whether incidence rates of pneumonia increased in primary care in the United Kingdom from 2002 to 2017, an open cohort study was conducted using electronic health records from the UK Clinical Practice Research Datalink. Clinically diagnosed pneumonia, influenza pneumonia, pleural infection and clinically suspected pneumonia, defined as chest infection treated with antibiotics, were evaluated. Age-standardised and age-specific rates were estimated. Joinpoint regression models were fitted and annual percentage changes (APC) were estimated. There were 70.7 million person-years of follow-up with 120 662 episodes of clinically diagnosed pneumonia, 1 831 005 of clinically suspected pneumonia, 23 814 episodes of influenza pneumonia and 2644 pleural infections over 16 years. The incidence of clinically diagnosed pneumonia increased from 1.50 per 1000 person-years in 2002 to 2.22 per 1000 in 2017. From 2010 to 2017, the APC in age-standardised incidence was 5.1% (95% confidence interval 3.4–6.9) compared with 0.3% (−0.6 to 1.2%) before 2010. Clinically suspected pneumonia incidence rates increased from 2002 to 2008 with an APC 3.8% (0.8–6.9) but decreased with an APC −4.9% (−6.7 to −3.1) from 2009 to 2017. Influenza pneumonia increased in the epidemic year of 2009. There was no overall trend in pleural infection. The results show that clinically diagnosed pneumonia has increased in primary care but there was a contemporaneous decline in recording of clinically suspected pneumonia or ‘chest infection’. Changes in disease labelling practice might partly account for these trends.

- **What is the key question?**
  Recent evidence suggests an increasing trend in pneumonia hospitalizations. This study analysed primary care electronic health records for more than 70 million patient years of follow-up to evaluate whether pneumonia incidence had increased in community settings.
- **What is the bottom line?**
  Clinically-diagnosed pneumonia increased from 2002 to 2017, with an acceleration in trend after 2011. There was a simultaneous decrease in the more frequent diagnosis of clinically-suspected pneumonia characterized as antibiotic-treated chest infection. This suggests that changes in diagnostic labelling may at least in part account for the apparent increase in clinically-diagnosed pneumonia.
- **Why read on?**
  The large study, including practices from throughout the UK, sheds new light on previous reports of increasing pneumonia hospitalisations. The study shows that in adults there have been divergent trends in different respiratory infection diagnoses with increasing clinicallydiagnosed pneumonia and reducing clinically-suspected pneumonia. This is in contrast to consistently decreasing lower respiratory infections in children.

## Introduction

With the advent of antibiotics, common but severe infections such as pneumonia could be effectively cured with access to effective antimicrobial treatment [1] but community acquired pneumonia (CAP) remains a major public health priority worldwide, disproportionally affecting younger and older populations [2–4]. As an ambulatory care sensitive condition, [5] pneumonia may be managed in primary care or may result in hospital admission, depending on assessment of severity [6]. Recently, increasing hospital admissions for pneumonia have been reported in several studies [2, 7, 8]. In the USA, Fry *et al*. [2] reported increasing pneumonia hospitalisation rates in people aged 65 years and older and suggested that increasing comorbidity might be a contributing factor. In England, analysis of hospital episode statistics showed increasing hospital admissions for pneumonia from 1997 to 2005 [7]. The increase did not appear to be fully explained by demographic change, nor by an increase in co-existing conditions [7]. In Oxfordshire, hospital admissions for CAP increased from 1998 to 2014, with a more rapid rate of increase after 2008 [8]. Trends were similar in all age groups [8]. Other studies also suggest that the rate of emergency hospital admissions for pneumonia is increasing [9, 10].

While accumulating evidence points to a growing burden of pneumonia on hospital services, this may not necessarily mean that the incidence of pneumonia disease is increasing in the general population. Patients with pneumonia treated in hospital settings may differ from those managed in community. The decision to admit to hospital may depend on a patient's general health condition and social circumstances, as well as severity of illness [11]. Pneumonia may complicate pre-existing illnesses that make individuals vulnerable to infectious pathogens [12, 13]. Pneumonia itself may contribute to deterioration in underlying or pre-existing medical conditions including heart failure [14] or coronary syndromes [15] for which hospitalisation may be indicated. Investigation of the epidemiology of respiratory syndromes in the community is therefore indicated.

In primary care, management of respiratory conditions may often be ‘symptom oriented’. Definitive diagnosis thorough confirmatory tests may be less readily available in comparison with secondary care. Family physicians in the UK use the Snomed CT [16] and Read Code classifications [17], which enable coding of comprehensive and detailed patient information including occupation, social circumstances, clinical symptoms and signs, clinical tests and use of medical services [16]. This is in contrast to the disease categorisations offered by the International Classification of Diseases (ICD) [18] used for coding of hospital episode statistics. Primary care classifications provide detailed and granular coding systems including a wider range of information to enable patient management in the community. In primary care, CAP cases may often be identified based on clinical features rather than from clinical investigations including radiology findings and bacteriological tests [11]. Less specific labels including ‘chest infection’ may be applied to clinically suspected pneumonia consultations. Clinical guidelines in the UK emphasise that the category of ‘chest infection’ may contain two general clinical scenarios: acute bronchitis and CAP, with antibiotics only being indicated for the latter [19]. Conversely, there may be no major differences between management recommendations for CAP and clinically suspected pneumonia labelled as ‘chest infection’ in terms of essential elements of treatment, severity assessment and referral principles in adult population [6, 19]. From a disease management perspective, adult CAP and antibiotic-treated chest infection cases might be labelled interchangeably during consultations in primary care.

To provide complementary information to hospital-based studies, this study aims to evaluate trends in the incidence of conditions managed as pneumonia in the community. In this study, CAP was included as ‘clinically diagnosed pneumonia’ whereas antibiotic-treated chest infection as ‘clinically suspected pneumonia’. Influenza pneumonia was included to assess whether secondary pneumonia complicating influenza has exerted a significant impact on pneumonia burden in the community [20, 21]. Pleural infection was also analysed because it shares a similar aetiology to pneumonia and may be associated with pneumonia severity, with recent studies documenting increasing pleural infection trends especially among children [22].

## Methods

### Data source and study design

A population-based cohort study was conducted in the UK Clinical Practice Research Datalink (CPRD). The CPRD is the world's largest database of primary care electronic health records, covering approximately 7% of the UK family practices [23]. More than 98% of the UK population were registered with a family practice [24]. The CPRD population is considered to be representative of the UK population; the database includes comprehensive data for drug prescriptions and diagnoses recorded in primary care, which have been shown to be valid in many studies [23, 24]. The CPRD comprises an open cohort of UK family practices and their registered patients. The present analysis included all eligible family practices contributing to CPRD, and data for all registered patients aged up to 100 years old, during 16 calendar years from the beginning of 2002 to the end of 2017.

### Cases

We included recorded diagnoses of pneumonia, antibiotic-treated chest infection, influenza pneumonia and pleural infection, including bacterial pleurisy and empyema. Read codes were reviewed and selected independently by two researchers with clinical and epidemiological backgrounds. Pneumonia was defined using Read codes associated with ‘pneumonia’ terms after excluding tuberculosis (TB), fungal and parasite pneumonia. Influenza pneumonia was evaluated as a separate group. The remaining pneumonia codes were grouped into a single category of ‘bacterial pneumonia’ after excluding non-infectious pneumonia codes e.g. bronchiolitis obliterans organising pneumonia [25]. This was consistent with the category of CAP being mainly used to refer to uncomplicated bacterial pneumonia in the general population. CAP was evaluated as ‘clinically diagnosed pneumonia’ in this study. Antibiotic-treated chest infection cases were identified when patients were recorded with ‘chest infections’ and received antibiotic prescription on the same day. ‘Antibiotics’ included antibacterial agents from chapter 5.1 of the British National Formulary without anti-viral, anti-TB, anti-leprosy and anti-fungal drugs [26]. Antibiotic-treated chest infection was then analysed as clinically suspected pneumonia. Empyema and bacterial pleurisy were analysed as pleural infection. Primary diagnoses were not differentiated when multiple diagnoses were recorded in a single consultation. Codes for the same condition in the same patient during a 90-day time-window were considered to represent a single episode.

### Statistical analysis

Person-time at risk was estimated for the CPRD registered population by year from 2002 to 2017 as a denominator. For each patient, we included time from the latest of the patient registration date, or the date the family practice began contributing data to CPRD, to the earliest of the patient end-of-registration, death date or the date the practice left CPRD. Incident events were considered as those recorded more than 1 year after the patient start-date to eliminate prevalent cases from any possible duplication of records during patient registration. Age-specific incidence rates were calculated using the age-groups 0–4 years, 5–14 years, then 10-year age-groups, up to 85 years and older. Data for participants aged more than 100 years were omitted as these are few in number and may be subject to data recording errors. Age- and sex-standardised incidence rates (ASRs) were calculated using the European standard population (2013 revision). To evaluate whether there had been any recent changes in trend, changes over calendar year were modelled by joinpoint regression analysis [27] using Joinpoint Trend Analysis software from the NIH National Cancer Institute [28]. The joinpoint method starts with a linear model and tests whether addition of joinpoints improves goodness of fit using Monte-Carlo permutation tests [27]. Annual percentage changes (APCs) were estimated to quantify the direction and slope of the trend in given period of time and average annual percent change (AAPC) was adopted to measure average rate changes across the whole study period.

### Sensitivity analyses

To evaluate whether changes in the family practice population influenced conclusions, we repeated analyses including only the 218 family practices that contributed data in each year of study from 2002 to 2017.

### Research ethics

The research protocol for this study was submitted to and approved by the Medicines and Healthcare Products Regulatory Agency (MHRA) Independent Scientific Advisory Committee (ISAC), Protocol 16_020. All patient medical records were anonymised before data received by researchers.

## Results

There were 550 family practices contributing to CPRD in 2002, increasing to 631 in 2007, before declining to 314 in 2017 (Table 1). There was a total of 4.2 million person-years of follow-up of registered patients aged up to 100 years in 2002, increasing to 5.03 million in 2008–2009, before declining to 2.5 million in 2017.

**Table 1.** Number of incidence events of pneumonia and related conditions

Year	Family practices	Person years	Clinically diagnosed pneumonia		Clinically suspected pneumonia		Influenza pneumonia		Pleural infection	
			Freq.	Rate^a^	Freq.	Rate^a^	Freq.	Rate^a^	Freq.	Rate^a^
2002	550	4 191 630	6291	1.50	99 256	23.68	956	0.23	136	0.03
2003	586	4 458 959	7189	1.61	116 781	26.19	1081	0.24	157	0.04
2004	612	4 767 931	7219	1.51	117 265	24.59	1264	0.27	157	0.03
2005	620	4 898 961	8184	1.67	139 785	28.53	1473	0.30	186	0.04
2006	626	4 956 288	7812	1.58	134 030	27.04	1454	0.29	192	0.04
2007	631	5 016 169	7798	1.55	151 411	30.18	1468	0.29	200	0.04
2008	627	5 025 191	8043	1.60	152 922	30.43	1500	0.30	207	0.04
2009	621	5 026 729	7977	1.59	133 848	26.63	6103	1.21	215	0.04
2010	613	4 967 771	8145	1.64	136 905	27.56	2110	0.42	210	0.04
2011	596	4 862 957	8141	1.67	127 963	26.31	1574	0.32	177	0.04
2012	580	4 805 309	8529	1.77	133 994	27.88	1309	0.27	196	0.04
2013	564	4 595 318	8376	1.82	112 730	24.53	1025	0.22	177	0.04
2014	530	4 201 387	7802	1.86	96 219	22.90	859	0.20	134	0.03
2015	462	3 605 006	7353	2.04	76 108	21.11	672	0.19	107	0.03
2016	371	2 861 175	6346	2.22	57 126	19.97	536	0.19	102	0.04
2017	314	2 455 307	5457	2.22	44 662	18.19	430	0.18	91	0.04

Figures are frequencies except where indicated.

a Rate per 1000 person years.

### Clinically diagnosed pneumonia

**Fig. 1.**
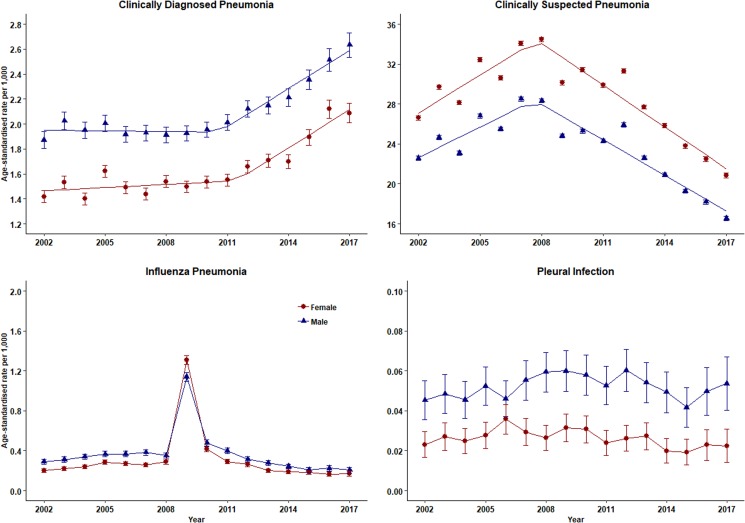
Trends in pneumonia and related conditions for both men (blue) and women (red) 2002–2017. Rates are per 1000 person-years.

**Fig. 2.**
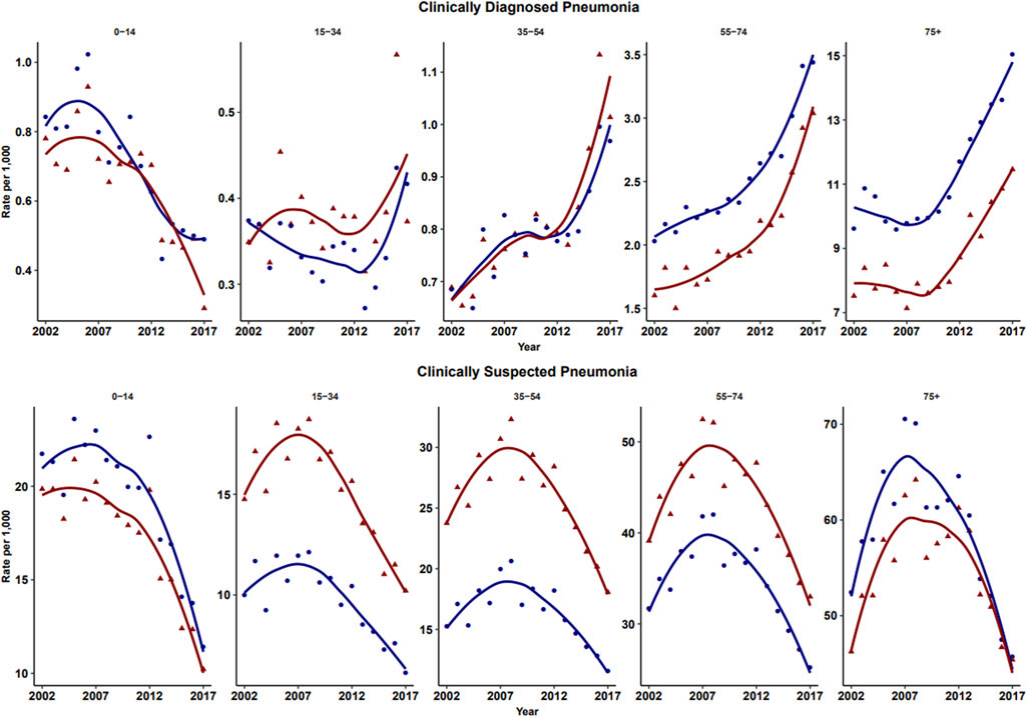
Age-specific rates for clinically diagnosed pneumonia and clinically suspected pneumonia for males (blue) and females (red). Rates are per 1000 person-years.

The number of episodes of clinically diagnosed pneumonia was between 5000 and 10 000 in each year of study (Table 1). Six codes accounted for 81% of all pneumonia episodes: ‘pneumonia due to unspecified organism’ (38%); ‘bronchopneumonia due to unspecified organism’ (14%); ‘history of pneumonia’ (12%); ‘community acquired pneumonia’ (8%); ‘lobar (pneumococcal) pneumonia’ (7%) and ‘lobar pneumonia due to unspecified organism’ (3%). The crude incidence rate of clinically diagnosed pneumonia increased from 1.50 (1.46–1.54) per 1000 patient years in 2002 to 1.64 (1.60–1.68) per 1000 in 2010, the rate then increased more rapidly to 2.22 (2.16–2.28) per 1000 in 2017. Figure 1 (top left panel) shows changes in the age-standardised rates of pneumonia for men and women separately with fitted lines from joinpoint regression. Trends were similar in men and women but clinically diagnosed pneumonia was more frequent in men. Table 2 presents estimates from the joinpoint regression model. The APC in age-standardised rate of clinically diagnosed pneumonia was 0.3% (95% confidence interval −0.6 to 1.2) per year from 2002 to 2010 but from 2010 to 2017 the APC was 5.1% (3.4–6.9) per year. The AAPC over the entire period was 2.4% (3.4–6.2) per year. Estimation of age-specific rates of pneumonia (Fig. 2) shows that clinically diagnosed pneumonia was reducing throughout the period in children aged under 15 years, while recorded clinically diagnosed pneumonia increased in adults, especially at older ages. In patients aged 15–54 years, rates of clinically diagnosed pneumonia were slightly higher in women than men but, but over the age of 55 years clinically diagnosed pneumonia was more frequent in men, especially at the oldest ages.

**Table 2.** Joinpoint regression estimates for APC

Condition	Measure	Year of joinpoint	APC (%) before joinpoint (95% CI)	APC (%) after joinpoint (95% CI)	Average APC (%) 2002 to 2017 (95% CI)
Clinically diagnosed pneumonia	Crude-rate	2010	0.4 (−0.7 to 1.5)	4.8 (3.4–6.2)	2.4 (3.4–6.2)
	ASR	2011	0.3 (−0.6 to 1.2)	5.1 (3.4–6.9)	2.2 (1.4–3.0)
Clinically suspected pneumonia	Crude-rate	2008	3.9 (0.9–7.1)	−4.7 (−6.6 to −2.9)	−1.4 (−2.8 to 0.1)
	ASR	2008	3.8 (0.8–6.9)	−4.9 (−6.7 to −3.1)	−1.5 (−2.9 to −0.1)

### Clinically suspected pneumonia

The annual number of cases of clinically suspected pneumonia ranged between 44 662 and 152 992. Two codes accounted for more than 99% of clinically suspected pneumonia: ‘chest infection not otherwise specified’ (61%) and ‘chest infection’ (39%). The crude rate of clinically suspected pneumonia was more than 10 times higher than for clinically diagnosed pneumonia, increasing from 23.7 in 2002 to 30.4 per 1000 in 2008 before declining to 18.2 per 1000 in 2017 (Table 1). Figure 1 (top right panel) shows that trends age-standardised rates of clinically suspected pneumonia were similar in men and women, but absolute rates were greater in women than in men. Joinpoint regression indicated a change in trend in 2008. The APC from 2002 to 2008 was 3.8% (0.8–6.9) per year compared with −4.9% (−6.7 to −3.1) per year after 2008. Changes in age-specific rates were generally consistent but clinically suspected pneumonia was more frequent in women from 15 to 74 years but more frequent in males during childhood and over the age of 75 years. The overall trend for all chest infection diagnoses was similar to clinically suspected pneumonia with the same turning point of 2008. While the proportions of all chest infections that were clinically suspected pneumonia increased steadily from 66% in 2002 to 88% in 2017 at an average APC of 1.6% (1.4–1.8) per year, suggesting that ‘chest infection’ not treated with antibiotics declined more rapidly than treated chest infection.

### Influenza pneumonia and pleural infection

Table 1 and Figure 1 present data for influenza pneumonia and pleural infection (including bacterial pleurisy and empyema). Rates of influenza pneumonia showed a peak in 2009 but remained low in other years. Rates of pleural infection were low and showed no consistent trend over time. Pleural infection was more frequent in men than women but there was no gender difference for influenza pneumonia.

### Sensitivity analysis

To evaluate whether attrition of family practices from CPRD, accounted for changes in coding, analyses were repeated using only data from 218 family practices that contributed data in every year from 2002 to 2017. In these 218 practices, the crude rate of clinically diagnosed pneumonia increased from 1.38 per 1000 in 2002 to 1.56 per 1000 in 2010 and then increased to 2.24 per 1000 in 2017 with an APC being 3.1% (1.9–4.2). For clinically suspected pneumonia, the crude rate increased from 20.8 per 1000 in 2002 to 29.7 per 1000 in 2008 at an APC of 4.0% (0.9–7.2) before declining to 18.0 per 1000 in 2017 at an average percentage of 5.1% (−6.6% to −3.4).

## Discussion

### Main findings

There was an increasing trend in clinically diagnosed pneumonia from 2002 onwards and this accelerated after 2011. This was unlikely to be due population ageing because similar trends were observed for age-standardised and crude incidence rates. Analysis of clinically suspected pneumonia showed that this syndrome was much more frequently recorded than clinically diagnosed pneumonia and incidence rates increased from 2002 to 2008 but decreased rapidly thereafter. Clinicians necessarily work with diagnostic disease classifications but pulmonary infections may represent graduated phenomena with varying degrees of bronchial inflammation an alveolar consolidation. This may contribute to diagnostic uncertainties and perhaps inconsistent selection of diagnostic terms. Given that ‘chest infection’ may not be a confident diagnosis, together with the volume of antibiotic-treated chest infection rates being considerably higher than that of diagnosed pneumonia, a small change in disease coding practice could lead to a shift from ‘chest infection’ recording to ‘pneumonia’ recording. Joinpoint regression analysis suggested that the decline in ‘chest infection’ recording began in 2008 slightly before the increase in ‘pneumonia’ recording from 2011. However, conditions managed as pneumonia were considered as relatively stable as none of the AAPCs have shown to be statistically significant from middle age and above. This would suggest that there was code drifting during clinical consultations when pneumonic infectious symptoms were presented among adult population with elder patient being more likely being diagnosed with pneumonia.

According to UK guidelines, adult ‘chest infection’ should be managed as CAP when pneumonia is suspected [20]. However, Petersen *et al*. [29] regarded pneumonia as a potential complication of chest infection in their electronic health records based study. If antibiotics are prescribed less frequently for chest infection it is possible that pneumonia might increase [30]. One study suggested that pneumonia might be more frequent at family practices that prescribe fewer antibiotics for respiratory infections [30]. Thus although it appears likely that changes in coding of respiratory infections account for observed trends, the present data do not exclude the possibility that changing management of ‘chest infections’ is leading to an increase in pneumonia incidence. Therefore, understanding the underlying reason for adopting certain disease labels such as ‘chest infection’ rather than confident disease diagnoses during routine healthcare in the community would contribute to understanding the challenges of diagnostic uncertainty in primary care settings.

In children, records of both clinically diagnosed pneumonia and clinically suspected pneumonia decreased. This could be explained by the introduction of Haemophilus Influenzae Type B vaccine in 1992 and pneumococcal conjugate 7 vaccine in 2006 into the UK childhood immunisation scheme [31]. In adults, rates of clinically diagnosed pneumonia increased while clinically suspected pneumonia decreased. Trends were generally similar in males and females but women between the ages of 15 and 74 years were more likely to be recorded with antibiotic-treated chest infection than men, but this distinction was not apparent for clinically diagnosed pneumonia. It is unclear whether this represents a disease classification preference or whether more severe cases tended to be found in men. Influenza pneumonia showed an increase in the epidemic year of 2009 [21] but overall low incidence of influenza pneumonia might result from the influenza vaccination schemes among both young and elder populations [31]. Pleural infection was analysed as a surrogate of severe pneumonia because recent evidence suggests an increase in incidence rate trends among children [22]. Our results showed that the incidence trends for pleural infection remained stable during the past 16 years.

### Comparison with previous studies

In contrast to previous studies on pneumonia burden using hospital admission data [7, 8], this study using electronic health records data with general practitioners being data recorders. Since pneumonia patients referred to secondary care may not necessarily be representative of all CAP, using primary care consultation data contributes to understanding pneumonia patients presenting and managed in primary care settings.

In National Institute for Health and Care Excellence (NICE) guidelines for both CAP and chest infection, together with British Thoracic Society recommendations for CAP management in adult patients, CRB-65 score (confusion, raised respiratory rate, low blood pressure and age 65 and above) is recommended to guide risk assessment and place of treatment [6, 19, 32]. This score identifies older age (⩾65 years old) as an independent risk score since being 65 years and above will automatically classify patients into an intermediate-risk group. This implies that more conservative management strategies have been applied to older pneumonia patients. This partially explained previous study findings that pneumonia patients in the community leading to hospitalisation were increasing in recent years among elder populations and there is no evidence that less severe patients were admitted to secondary care.

### Strengths and limitations

This population-based study analysed healthcare records to outline the range of conditions that were managed as pneumonia in the community. The 16-year timeframe provided sufficient data to estimate disease trends over a substantial study period. The study included all eligible practices and patients contributing health care information to CPRD more than 1 year. There were 320 practices participating the database by the end of 2017. Previous studies have shown the completeness and data of high quality in CPRD. The large sample size of research cohort was sufficient for depicting the trends for clinically diagnosed pneumonia, clinically suspected pneumonia, even low incidence conditions such as influenza pneumonia and pleural infections.

CPRD has not released free text information since 2013, which would enable us to examine diagnostic information documented in free text records rather than coded. But, we consider that clinicians would record relevant conditions such as pneumonia or chest infection especially when clinical discretion leads to antibiotic treatment. Also, health care information in CPRD made disease severity assessment unfeasible, therefore, we could not confidently determine whether case severity influenced coding practices or place of treatment. Also, we did not capture data from out-of-hour services, walk-in centre consultations and emergency care.

This study was derived from a universal health care coverage system where most common conditions are managed in primary care settings, implications generated from this study would mainly apply to similar systems but not where health care insurance plays an essential role or referral thresholds from primary care to secondary care vary extensively compared with that in the UK.

## Conclusion

Clinically diagnosed pneumonia is increasing over time in the UK. This trend could not be fully explained by ageing population, changes in coding practice or alternative diagnosis. Age-specific trends were divergent with decreasing trends in children but increasing in older adults. Respiratory conditions managed as pneumonia in family practice were decreasing slightly over time, which was more likely due to more conservative antibiotic prescribing strategies. Research to reduce diagnostic uncertainty would contribute to improving antibiotic stewardship in the community.
